# Approaching the bi-objective critical node detection problem with a smart initialization-based evolutionary algorithm

**DOI:** 10.7717/peerj-cs.750

**Published:** 2021-10-21

**Authors:** Eliézer Béczi, Noémi Gaskó

**Affiliations:** Babeş-Bolyai University of Cluj-Napoca, Cluj-Napoca, Romania

**Keywords:** Complex networks, Critical nodes, Bi-objective critical node detection, Multi-objective algorithms, Memetic algorithms

## Abstract

Determining the critical nodes in a complex network is an essential computation problem. Several variants of this problem have emerged due to its wide applicability in network analysis. In this article we study the bi-objective critical node detection problem (BOCNDP), which is a new variant of the well-known critical node detection problem, optimizing two objectives at the same time: maximizing the number of connected components and minimizing the variance of their cardinalities. Evolutionary multi-objective algorithms (EMOA) are a straightforward choice to solve this type of problem. We propose three different smart initialization strategies which can be incorporated into any EMOA. These initialization strategies take into account the basic properties of the networks. They are based on the highest degree, random walk (RW) and depth-first search. Numerical experiments were conducted on synthetic and real-world network data. The three different initialization types significantly improve the performance of the EMOA.

## Introduction

In recent years, complex networks have received a lot of attention due to their applicability in various domains. Several optimization problems were studied within complex networks like community detection (Fortunato, 2010), maximal influence node detection (Kempe, Kleinberg & Tardos, 2003) and link prediction (Liben-Nowell & Kleinberg, 2007). All of the aforementioned problems reveal major insights into the networks studied.

Identifying critical nodes (the critical node detection problem, or CNDP) in a complex network is a crucial task. The base problem consists of minimizing pairwise connectivity by removing a subset of *K* nodes in a given graph. In Arulselvan et al. (2009) it was proven to be an NP-hard problem.

The general formulation of the problem is Lalou, Tahraoui & Kheddouci (2018): given a *G* = (*V*, *E*) graph and a connectivity metric *λ*, find the set of nodes *S*⊆*V* such that *G*[*V*∖*S*] satisfies the metric *λ*. This metric is usually defined as an objective function that needs to be optimized (for example, maximize the number of components, minimize the component size, etc.).

CNDP has a wide field of applicability, for example in social network analysis (Fan & Pardalos, 2010), epidemic control (Tao, Zhongqian & Binghong, 2006), network immunization (Kuhlman et al., 2010) and biological networks (Liu et al., 2020). Several algorithms were designed for the CNDP. The majority of the exact methods are based on the integer linear programming formulation of the problem (Summa, Grosso & Locatelli, 2012). In Addis, Summa & Grosso (2013), a dynamic programming approach is proposed for a special class of graphs. As approximation algorithms, we can mention, for example, a simulated annealing algorithm (Ventresca, 2012). A thorough survey of existing methods for the CNDP can be found in Lalou, Tahraoui & Kheddouci (2018).

The CNDP has several variants exploring the connectivity metric *λ*. Other variants with constraints were introduced, such as the cardinality constrained critical node detection problem (CC-CNDP) (Arulselvan et al., 2011) and the component-cardinality-constrained critical node problem(3C-CNDP) (Lalou, Tahraoui & Kheddouci, 2016). One of the existing bi-objective variants of the CNDP is proposed in Li et al. (2019). In this variant the cost of removing the node counts. Another bi-objective variant proposed in Ventresca, Harrison & Ombuki-Berman (2018) is the base of our study (described in ‘The Bi-objective Critical Node Detection Problem’).

Evolutionary algorithms are powerful tools in optimization problems. Multi-objective optimization problems involve multiple objective functions which need to be optimized at the same time, so they can be used in real-world optimization problems. Because the BOCNDP is an NP-hard problem (Ventresca, Harrison & Ombuki-Berman, 2018), the use of the evolutionary algorithms is straightforward. To increase the performance of evolutionary algorithms, several techniques were designed, for example, hybridization, a special case of memetic algorithms which incorporates a local search in the initialization phase. Kazimipour, Li & Qin (2014) emphasizes the importance of population initialization techniques, introduces a new categorization, and mentions some concrete examples.

Due to the wide applicability of the critical node detection problem, this article introduces new smart initialization strategies that can be incorporated into any multi-objective optimization algorithm that treats the BOCNDP to increase its performance. These strategies can be used in other variants of the CNDP, or even for other computationally graphed theoretical problems because they take into account structural information about the network.

To summarise, the main goal of this article is as follows:

• a smart initialization which is based on a depth-first search: nodes lying on a path are chosen to be in the initial population;
• a random walk-based smart initialization strategy: a random walk is simulated on the graph, and nodes that appear more times in the walk are considered more important;
• a degree-based smart initialization strategy: nodes with a higher degree are more likely to be chosen in the initial population;
• statistical analysis of the three smart initialization strategies introduced here and their comparison with random initialization.

The rest of the article is organized as follows: In the second section, we describe the bi-objective critical node detection problem and the existing solving algorithms. In the third section, we present the proposed initialization algorithms. The next section describes the numerical experiments. The article ends with conclusions and further work.

## The bi-objective critical node detection problem

Let *G* = (*V*, *E*) be an undirected graph, where *V* is the set of nodes, and *E* is the set of edges.

Let *G* = (*V*, *E*) be an undirected graph, where *V* is the set of nodes, and *E* is the set of edges. The bi-objective critical node detection problem was proposed in Ventresca, Harrison & Ombuki-Berman (2018) and consists of finding a fixed number of *k* nodes, which, if deleted from graph *G*, will optimize the following two objectives:

1. maximize the number of connected components;
2. minimize the variance of the cardinality of the connected components.

Formally the objectives are the following:

(1) \begin{eqnarray*}\max \nolimits \hspace*{10.00002pt}{|}H{|},\end{eqnarray*}

(2) \begin{eqnarray*}\min \nolimits \hspace*{10.00002pt}var(H),\end{eqnarray*}

(3) \begin{eqnarray*}\text{such that}\sum _{i\in S}{w}_{i}\leq W,\end{eqnarray*}

where *w*_*i*_ are the weights associated to the vertices of the graph and *W* > 0 is a constraint, *H* denotes $G \left[ V\setminus S \right] $ the set of the connected components and *var*(*H*) denotes the variance of the cardinality of the connected components and can be calculated in the following way:

(4) \begin{eqnarray*} \frac{1}{{|}H{|}} \sum _{h\in H}{ \left( {|}h{|}- \frac{{n}^{\ast }}{{|}H{|}} \right) }^{2},\end{eqnarray*}

where *n*^∗^ = ∑_*h*∈*H*_|*h*| is the number of nodes in $G \left[ V\setminus S \right] $. The BOCNDP is different from the CNDP (Ventresca, Harrison & Ombuki-Berman, 2018).

**Figure 1:**
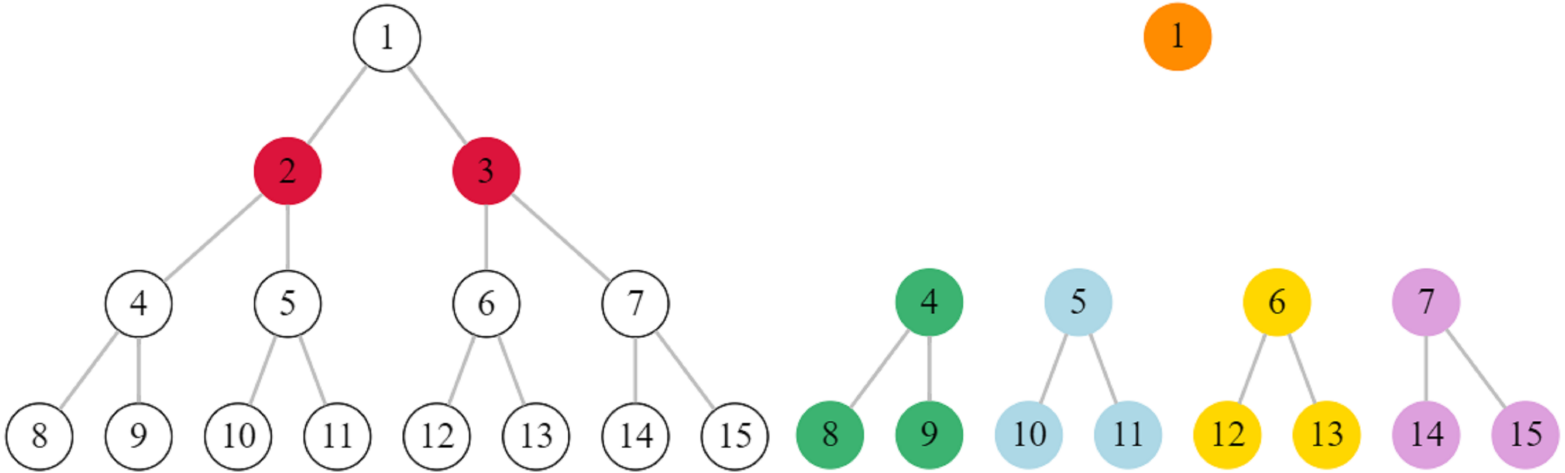
A small graph with 15 nodes. If we delete the second and the third nodes (left), the graph will have 5 connected components (right).

Let us consider a simple example, the graph presented in Fig. 1. If we need to identify *k* = 2 critical nodes, then $S= \left\{ 2,3 \right\} $ is the optimal solution. The $G \left[ V\setminus S \right] $ will have 5 components, |*H*| = 5 and

\begin{eqnarray*}var(H)= \frac{1}{5} \cdot \left[ { \left( 1- \frac{13}{5} \right) }^{2}+4\cdot { \left( 3- \frac{13}{5} \right) }^{2} \right] = \frac{16}{25} =0.64. \end{eqnarray*}

Because the BOCNDP is a relatively new problem formulation, the literature is not rich in proposed algorithms. In Ventresca, Harrison & Ombuki-Berman (2018), six existing multi-objective algorithms are used to solve the BOCNDP. In Li et al. (2019), a different variant of the BOCNDP, called Bi-CNDP, is introduced and studied with decomposition-based multi-objective evolutionary algorithms.

## Evolutionary computation method

Evolutionary algorithms are powerful optimization tools, especially in multi-objective optimization problems. To increase the performance of these algorithms, hybrid versions are designed and analysed. Smart initialization of the population of an evolutionary algorithm can increase the performance of the algorithm significantly (Maaranen, Miettinen & Penttinen, 2007).

We present three strategies that can be used in the initialization phase of any multi-objective algorithm. The first one is based on a depth search algorithm, outlined in the algorithm 1. A depth-first search (DFS) algorithm is started with a random initial node, and every *x*th element will be added to the chromosome, where $x= \frac{{|}V{|}}{k} $, |*V*| is the number of nodes and *k* is the number of critical nodes.

The second initialization method is based on the degree distribution of the nodes. The first *x* nodes with the highest degree are set in the chromosome, and the rest of the *k* − *x* nodes are selected randomly, to preserve the stochastic nature of the initialization (Algorithm 2).

The third method is based on a random walk. We start the walk from a random node, *t* is the length of the walk and *p*_*r*_ is the probability to restart the walk. In each step, the decision is to continue the walk or to restart. If we fail to walk through *k* different nodes, the algorithm will restart from another initial point. In the walk, we keep counting how many times a node appears. The more times it appears, the higher the probability it is a gene in the chromosome. The main steps are presented in the algorithm 3.

These initialization strategies can be used in any kind of multi-objective evolutionary algorithm. The outline of the smart initialization-based algorithm is depicted in the algorithm 4.

 
_______________________ 
Algorithm 1 Depth-first search solution generator (DFS)___________________________ 
Require: G,k,x 
 1:  start ← SELECT(V ) 
  2:  S ← DFS(G,start) 
  3:  return S [::x]                                              ⊳ Take every xth element    


 
_______________________________________________________________________________________________________ 
Algorithm 2 Degree solution generator (Deg)__________________________________________ 
Require: G,k,x 
 1:  V ′ ← SORTED(V )    ⊳ Sort nodes according to their degree in DESC order 
  2:  S ← V ′ [:x]                         ⊳ Take first x nodes with the highest degree 
  3:  while |S| < k do 
 4:       node ← SELECT(V ′) 
  5:       if node / ∈ S then 
 6:            S ← S ∪{node} 
 7:       end if 
 8:  end while 
 9:  SHUFFLE(S) 
10:  return S_________________________________________________________________________ 


 
________________________________________________________________________________________ 
Algorithm 3 Random walk solution generator (RW)_________________________________ 
Require: G,k,t,pr 
  1:  visited ←∅ 
 2:  while True do 
 3:       core ← SELECT(V ) 
  4:       current ← core 
 5:       for i ← 1,t + 1 do 
 6:            if current ∈ visited then 
 7:                  visited[current] ← visited[current] + 1 
  8:            else 
 9:                  visited[current] ← 1 
10:            end if 
11:            restart ← RAND INT(1,100) 
12:            if restart ≤ pr then 
13:                  current ← core 
14:            else 
15:                  neighbors ← NEIGHBORS(G,current)⊳ Neighbors of the current 
    node 
16:                  current ← SELECT(neighbors) 
17:            end if 
18:       end for 
19:       if |visited|≥ k then 
20:            break 
21:       else 
22:            visited ←∅ 
23:       end if 
24:  end while 
25:  SORT(visited)      ⊳ Sort nodes in visited according to visits paid in DESC 
     order 
26:  return visited[:k]                         ⊳ Take the first k most visited nodes    


 
_______________________________________________________________________________________________________ 
Algorithm 4 Evolutionary algorithm with smart initialization_____________________ 
Require: G,k 
 1:  initialize1 population Ps; 
  2:  run  a  multi-objective  Pareto  based  optimization  algorithm2,   where 
     Pinitial = Ps; 
  3:  return Pareto front________________________________________________________________________ 
1for the initialization we use:  random initialization, depth-first search, degree, 
random walk; 
2e.g., NSGA-II, SPEA__________________________________________________________________________  


## Numerical experiments

### Benchmarks

#### Synthetic data

We use the synthetic graph set proposed in Ventresca (2012). The benchmark set contains four different types of graphs: Barabási-Albert (BA), Erds-Rényi (ER), Forest-fire (FF) and Watts–Strogatz (WS). Barabási-Albert graphs are scale-free networks, using a preferential attachment mechanism and some high degree nodes (hubs). Erds-Rényi graphs are random networks in which each link between nodes is generated randomly based on a probability. Forest-fire graphs are random graphs with a preferential attachment mechanism. Watts–Strogatz graphs are random graphs with short average path lengths, so they have a dense structure.

Table 1 presents some basic properties of the benchmarks used: number of nodes (|*V*|), number of edges (|*E*|), the number of critical nodes (*k*), average degree (〈*d*〉), density of the graph (*ρ*), and average path length (*l*_*G*_).

**Table 1:** Benchmark test graphs and basic properties.

Graph	|*V*|	|*E*|	*k*	〈*d*〉	*ρ*	*l*_*G*_
BA500	500	499	50	1.996	0.004	5.663
BA1000	1000	999	75	1.998	0.002	6.045
BA2500	2500	2499	100	1.999	0.001	6.901
BA5000	5000	4999	150	2.000	0.000	8.380
ER250	235	350	50	2.979	0.013	5.338
ER500	466	700	80	3.004	0.006	5.973
ER1000	941	1400	140	2.976	0.003	6.558
ER2500	2344	3500	200	2.986	0.001	7.516
FF250	250	514	50	4.112	0.017	4.816
FF500	500	828	110	3.312	0.007	6.026
FF1000	1000	1817	150	3.634	0.004	6.173
FF2000	2000	3413	200	3.413	0.002	7.587
WS250	250	1246	70	9.968	0.040	3.327
WS500	500	1496	125	5.984	0.012	5.304
WS1000	1000	4996	200	9.992	0.010	4.444
WS1500	1500	4498	265	5.997	0.004	7.554

**Real dataset** Nine real datasets are used for the numeric experiments. The real datasets come from different areas: transportation networks (USAir97, TrainsRome, EUFlights), biological networks (Bovine, EColi, HumanDis), social networks (Oclinks, Facebook), and an electric network (Circuit). The size of the graphs varies from 121 to 4039 nodes. The density of the networks varies from 0.008 to 0.044 and the average path length is from 2.622 to 43.496. The basic properties of the networks are outlined in Table 2.

**Table 2:** Real graphs and basic properties.

Graph	|*V*|	|*E*|	*k*	〈*d*〉	*ρ*	*l*_*G*_	Ref.
Bovine	121	190	12	3.140	0.026	2.861	Reimand et al. (2008)
Circuit	252	399	25	3.167	0.012	5.806	Milo et al. (2004)
EColi	328	456	15	2.780	0.008	4.834	Yang, Huang & Lai (2008)
USAir97	332	2126	33	12.807	0.038	2.738	Rossi & Ahmed (2015)
HumanDis	516	1188	52	4.605	0.008	6.509	Goh et al. (2007)
TrainsRome	255	272	26	2.133	0.008	43.496	Cacchiani, Caprara & Toth (2010)
EUFlights	1191	31610	119	53.081	0.044	2.622	Opsahl (2011)
Oclinks	1839	13838	190	14.574	0.008	3.055	Opsahl & Panzarasa (2009)
Facebook	4039	88234	404	43.691	0.010	3.693	Leskovec & Krevl (2014)

### Statistical analysis of the smart initialization strategies

To analyse the behaviour of the initialization strategies introduced, we generated 100 independent solutions and calculated the values of |*H*| and *var*(*H*). A statistical test was conducted to mark the differences between the methods. Table 3 presents the results. In almost all cases, the degree-based initialization outperformed the other strategies. Almost in all cases the degree based initialization outperformed the other strategies, but all of them outperformed the random initialization.

**Table 3:** Average value ± standard deviation of the for all datasets over 100 independent runs.

Graph	Random		DFS		Deg		RW	
	|*H*|	*var*(*H*)	|*H*|	*var*(*H*)	|*H*|	*var*(*H*)	|*H*|	*var*(*H*)
BA500	49.36 ± 21.74	3097.82 ± 2112.76	41.15 ± 11.15	3695.26 ± 1462.78	244.28 ± 18.65^*^	3.05 ± 1.36^*^	103.81 ± 32.89	441.45 ± 2365.44
BA1000	73.31 ± 36.73	10002.81 ± 5993.79	60.37 ± 24.97	12836.08 ± 5227.38	474.91 ± 29.23^*^	4.21 ± 1.51^*^	176.79 ± 61.87	1652.69 ± 4710.80
BA2500	98.03 ± 39.55	54123.97 ± 24512.87	89.26 ± 32.17	60285.58 ± 24166.17	860.18 ± 63.00^*^	18.64 ± 6.30^*^	252.58 ± 97.64	9704.90 ± 18968.13
BA5000	139.40 ± 34.40	148039.52 ± 47033.66	142.23 ± 42.43	152707.13 ± 54715.66	1533.22 ± 104.56^*^	26.19 ± 6.59^*^	337.75 ± 138.00	30651.02 ± 37103.39
ER250	14.38 ± 3.15	1827.15 ± 497.41	19.33 ± 3.90	1154.09 ± 1274.76	26.00 ± 4.17^*^	765.19 ± 220.02^*^	12.81 ± 2.55	2090.96 ± 485.71
ER500	21.02 ± 3.67	5936.26 ± 1206.28	24.69 ± 4.62	4796.56 ± 3592.21	36.89 ± 5.12^*^	3007.85 ± 584.29^*^	18.31 ± 3.49	6946.33 ± 1509.55
ER1000	42.20 ± 5.01	12845.73 ± 1675.54	46.76 ± 7.35	11765.73 ± 7683.25	77.09 ± 8.15^*^	5947.44 ± 927.67^*^	38.99 ± 4.92	13974.21 ± 2125.06
ER2500	55.97 ± 7.00	75946.14 ± 10701.36	56.94 ± 5.94	73241.04 ± 7760.67	105.14 ± 10.41^*^	38069.15 ± 4333.11^*^	50.61 ± 6.81	84401.80 ± 12291.82
FF250	20.12 ± 4.54	1360.18 ± 518.16	21.27 ± 3.95	1164.85 ± 315.27	51.89 ± 7.85^*^	36.68 ± 41.49^*^	38.25 ± 9.23	221.69 ± 248.40
FF500	53.97 ± 7.37	1449.86 ± 401.19	66.10 ± 7.41	895.44 ± 214.06	130.62 ± 12.81^*^	9.31 ± 3.27^*^	77.74 ± 12.84	164.61 ± 180.47
FF1000	68.47 ± 9.04	7344.12 ± 1498.93	79.71 ± 8.85	5491.09 ± 966.88	173.03 ± 15.29^*^	49.80 ± 20.63^*^	110.50 ± 19.33	1402.81 ± 1121.88
FF2000	97.52 ± 10.87	24888.22 ± 3761.80	99.17 ± 10.83	24149.41 ± 3287.01	270.92 ± 31.20^*^	121.23 ± 67.59^*^	135.65 ± 31.73	6694.04 ± 8302.33
WS250	1.00 ± 0.00	0.00 ± 0.00^*^	1.00 ± 0.00	0.00 ± 0.00^*^	1.00 ± 0.00	0.00 ± 0.00^*^	1.09 ± 0.29^*^	711.12 ± 2272.67
WS500	1.21 ± 0.50	5633.93 ± 12549.71	1.01 ± 0.10	318.62 ± 3186.22^*^	2.08 ± 1.00^*^	20254.58 ± 14955.09	1.78 ± 0.98	15701.24 ± 16313.11
WS1000	1.00 ± 0.00	0.00 ± 0.00^*^	1.00 ± 0.00	0.00 ± 0.00^*^	1.01 ± 0.10	1592.01 ± 15920.10	1.35 ± 0.61^*^	44980.28 ± 70915.49
WS1500	1.18 ± 0.41	63783.38 ± 141708.20	1.11 ± 0.31	38209.49 ± 109239.42	2.47 ± 1.12	263515.70 ± 136371.28^*^	3.54 ± 1.48^*^	276008.24 ± 98915.66
Bovine	6.58 ± 10.11	1218.83 ± 1179.19	2.63 ± 4.59	1171.03 ± 1351.67	94.91 ± 5.24^*^	1.02 ± 0.94^*^	87.56 ± 11.08	4.82 ± 8.35
Circuit	4.03 ± 1.66	9182.68 ± 2622.23	3.69 ± 1.56	9007.61 ± 3059.59	7.72 ± 2.50^*^	4853.82 ± 2099.33^*^	4.50 ± 1.82	8127.30 ± 2820.42
EColi	9.24 ± 8.12	12020.10 ± 7415.24	11.01 ± 8.97	8373.55 ± 6239.28	103.58 ± 16.47^*^	189.90 ± 118.58^*^	45.93 ± 22.61	2568.58 ± 3649.19
USAir97	7.53 ± 4.84	11035.84 ± 5756.78	7.55 ± 5.48	12066.09 ± 6284.24	54.00 ± 5.28	1038.70 ± 160.83	62.99 ± 7.90^*^	794.91 ± 451.27^*^
HumanDis	18.53 ± 5.79	9095.70 ± 4116.18	20.01 ± 5.46	7639.00 ± 3162.30	76.58 ± 8.19^*^	160.28 ± 80.79^*^	30.62 ± 10.36	4623.64 ± 3005.66
TrainsRome	17.85 ± 1.79	273.49 ± 140.99	21.05 ± 0.80^*^	64.73 ± 37.06^*^	21.32 ± 1.86^*^	184.62 ± 55.62	1.87 ± 0.82	5223.65 ± 5085.48
EUFlights	18.89 ± 9.15	66944.36 ± 28610.83	16.98 ± 7.30	73118.63 ± 34156.37	25.84 ± 5.72	42239.73 ± 8314.82	44.30 ± 15.39^*^	25945.04 ± 8952.33^*^
Oclinks	41.93 ± 12.59	70563.01 ± 22486.35	45.30 ± 10.31	61325.98 ± 15792.50	294.12 ± 23.86	6728.72 ± 789.91	349.02 ± 19.11^*^	5189.44 ± 466.75^*^
Facebook	10.76 ± 9.60	929504.70 ± 865354.95	15.64 ± 11.70	642310.12 ± 473911.43	75.16 ± 6.14^*^	164435.23 ± 15504.56^*^	40.67 ± 18.65	382094.53 ± 230206.80

**Notes.**

* An asterisk (*) indicates the best results based on a Wilcoxon sign-rank test (separately for |*H*| and *var*(*H*)).

### Algorithm

For the numerical experiments, we used the NSGA-II (Deb et al., 2002) algorithm within the Platypus (https://github.com/quaquel/Platypus, last accessed 3/12/2019) framework. NSGA-II is a multi-objective evolutionary algorithm in which every member of the population is sorted according to the level of non-domination. To maintain diversity, a crowding distance is applied.

### Parameter setting

For the numerical experiments, parameters of the NSGA-II algorithm are the default values of the Platypus framework, with a total evaluation number of 10000. All the weights of the nodes are set to 1, and *W* equals the number of nodes. Parameters of the smart initialization strategies are as follows: for the DFS, *x* is the number of nodes divided by the value of *k*; for the random walk, the number of steps is 10000 and the probability of restart is 0.2; and for the Deg algorithm, $x= \frac{k}{3} $.

### Performance evaluation

For the performance evaluation, we use the hypervolume indicator (Zitzler & Thiele, 1998; Zitzler & Thiele, 1999), a popular measure for multi-objective optimization algorithms. The hypervolume indicator measures the volume of the region of the dominated points in the objective space bounded by a reference point.

## Results and discussion

**Figure 2:**
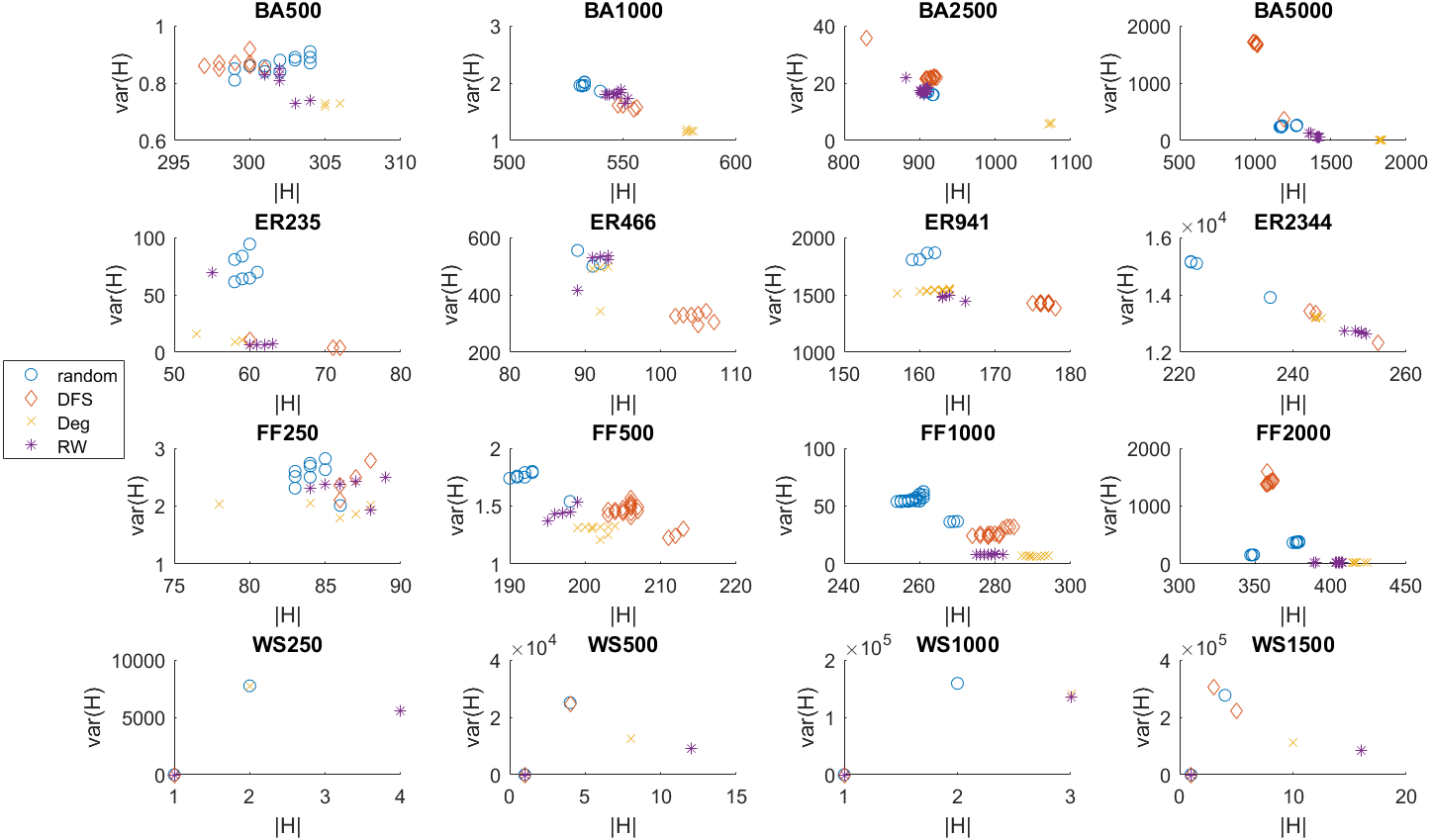
Pareto front obtained in a single run for the 16 synthetic benchmark problems.

In the case of synthetic benchmarks, we conducted ten independent runs for each initialization strategy (depth-first search, degree-based, random walk) and made comparisons with random initialization. Table 4 presents the mean values and the standard deviation of the hypervolume indicators. For a reference point, we set the nadir point of all unified Pareto fronts. We conducted a Wilcoxon sign rank nonparametric test for the hypervolume indicator reported by each method. The Wilcoxon sign rank assesses if there is a significant difference between the two sample means. An (*) is used to indicate the statistical significance of differences. All initialization strategies which are not statistically different from the best one are marked. Figure 2 presents the Pareto front obtained within a single run.

Table 5 describes the mean value and standard deviation obtained for the real datasets. Best results are marked with an (*).

Based on the results, we can draw a general conclusion for the synthetic benchmarks about the best initialization strategy. The structure of the graph determines which initialization is worth using, but based on the numerical experiments, all of them give better results than the random initialization. In the case of Barabási-Albert graphs, which contain hubs, the degree-based initialization gets the best result. Erds-Rényi graphs are random graphs, in which case the depth-first search algorithm seems to be best. For Forest-fire graphs, which are random graphs, the three proposed initialization types gave almost the same result. The Watts–Strogatz graphs have a dense structure, and the best results were provided by the random walk-based algorithm.

**Table 4:** Average value ± standard deviation of the hypervolume indicator for the synthetic benchmarks.

Graph	Random	DFS	Deg	RW
BA500	15.65 ± 4.37	13.03 ± 5.37	28.05 ± 2.62^*^	20.72 ± 4.37
BA1000	36.54 ± 21.58	58.38 ± 24.17	154.27 ± 10.54^*^	68.21 ± 26.51
BA2500	6138.01 ± 1701.47	2924.91 ± 2828.77	18607.82 ± 364.64^*^	8643.83 ± 1993.09
BA5000	1078479.10 ± 319614.94	449069.24 ± 396267.77	3409301.92 ± 47950.42^*^	1706543.15 ± 182869.56
ER250	738.68 ± 217.41	2387.05 ± 342.45^*^	1162.31 ± 252.49	1204.39 ± 319.17
ER500	1607.75 ± 920.84	8045.09 ± 1848.71^*^	2762.01 ± 1572.09	2644.67 ± 919.06
ER1000	1133.40 ± 949.75	9407.93 ± 4141.55^*^	6385.57 ± 3487.82^*^	5176.12 ± 1499.51
ER2000	39323.23 ± 24560.07	65449.57 ± 34152.98^*^	56123.63 ± 12683.44^*^	72259.64 ± 26198.93^*^
FF250	38.01 ± 6.84	36.34 ± 12.26^*^	41.88 ± 5.39^*^	45.61 ± 7.52^*^
FF500	24.51 ± 8.19	51.42 ± 9.68^*^	37.72 ± 4.93	32.98 ± 6.86
FF1000	1402.85 ± 599.70	1718.09 ± 647.33	3274.96 ± 387.73^*^	3194.72 ± 409.27^*^
FF2000	30560.10 ± 20460.26	16206.67 ± 13027.87	136548.86 ± 12378.66^*^	107945.88 ± 12371.82
WS250	8132.90 ± 268.63	9577.11 ± 1390.61	8495.50 ± 636.66	16193.42 ± 3306.69^*^
WS500	48526.56 ± 14388.26	35879.19 ± 8263.24	110772.29 ± 31047.79	203301.59 ± 30420.58^*^
WS1000	159361.50 ± 503.68	159202.10 ± 0.32	162811.19 ± 11412.20	337726.99 ± 90573.11^*^
WS1500	484347.05 ± 140137.66	708776.63 ± 223377.92	1923479.18 ± 351573.85	5058526.43 ± 1741808.94^*^

**Notes.**

* An asterisk (*) indicates the best results which are based on a Wilcoxon sign-rank test.

**Table 5:** Average value ± standard deviation of the hypervolume indicator for the real datasets.

Graph	Random	DFS	Deg	RW
Bovine	275259.54 ± 57721.31	263669.06 ± 62622.52	4524666.52 ± 3.17^*^	3148983.90 ± 3.24
Circuit	77, 116.15 ± 29, 312.55	57695.87 ± 31444.12	1419610.33 ± 95327.38^*^	1086345.36 ± 77486.85
EColi	498186.23 ± 84010.17	368140.36 ± 172105.46	9742206.66 ± 165771.44^*^	6820902.13 ± 71640.00
USAir	193223.67 ± 115193.40	361106.95 ± 137617.76	5895064.90 ± 123050.07^*^	4010784.11 ± 95995.05
HumanDis	667579.05 ± 18327.70	762880.99 ± 14569.84	7944126.85 ± 156209.02^*^	5659398.93 ± 72630.76
TrainsRome	138525.07 ± 5559.37	154874.11 ± 4437.33	1727489.76 ± 41083.54^*^	1017365.41 ± 59551.06
EUFlights	59657.61 ± 34396.03	111198.59 ± 28682.44	9274581.78 ± 279065.65^*^	6373823.26 ± 143830.93
OClinks	291207.54 ± 92826.46	313517.98 ± 163921.22	26002131.74 ± 584868.91^*^	18601730.00 ± 296910.80
Facebook	4920.00 ± 0.00	5273.00 ± 0.00	1031865.98 ± 1185922.59	1409016.39 ± 817382.53^*^

**Notes.**

* An asterisk (*) indicates the best results which are based on a Wilcoxon sign-rank test.

In the case of the real networks, all three proposed initialization strategies outperformed the random initialization. In most cases, the degree-based initialization seemed to give the best results.

## Conclusions and further work

In this paper, we propose three smart population initialization methods for the BOCNDP problem. Numerical experiments show the effectiveness of the proposed approaches. All three methods outperformed the traditional random initialization.

As further work, other initialization strategies will be investigated and an adaptive algorithm can be developed to find the best initialization, taking into account the basic properties of the graph.

## Supplemental Information

10.7717/peerj-cs.750/supp-1 Supplemental Information 1 Pareto front of the benchmarks obtained with the four methods (depth-first search, high degree, random walk and random)

## Additional Information and Declarations

### Data Availability

The following information was supplied regarding data availability: The code and data are available at GitHub: https://github.com/Eliezer-Beczi/CNDP. The source of networks used for analysis are described in the article.
